# Increased phosphatidylethanolamine N-methyltransferase gene expression in non-small-cell lung cancer tissue predicts shorter patient survival

**DOI:** 10.3892/ol.2014.2035

**Published:** 2014-04-04

**Authors:** DAVID ZINRAJH, GERD HÖRL, GÜNTHER JÜRGENS, JANJA MARC, MIHA SOK, DARKO CERNE

**Affiliations:** 1 Chair of Clinical Biochemistry, Faculty of Pharmacy, University of Ljubljana, SI-1000 Ljubljana, Slovenia; 2 Department of Physiological Chemistry, Center for Physiological Medicine, Medical University of Graz, A-8010 Graz, Austria; 3 Department of Thoracic Surgery, University Medical Centre Ljubljana, SI-1000 Ljubljana, Slovenia

**Keywords:** phosphatidylethanolamine N-methyltransferase, lung cancer tissue, gene expression, survival analysis, fatty acid synthase, lipoprotein lipase

## Abstract

Lipid mobilization is of great importance for tumor growth and studies have suggested that cancer cells exhibit abnormal choline phospholipid metabolism. In the present study, we hypothesized that phosphatidylethanolamine N-methyltransferase (PEMT) gene expression is increased in non-small-cell lung cancer (NSCLC) tissues and that increased gene expression acts as a predictor of shorter patient survival. Forty-two consecutive patients with resected NSCLC were enrolled in this study. Paired samples of lung cancer tissues and adjacent non-cancer lung tissues were collected from resected specimens for the estimation of *PEMT* expression. SYBR Green-based real-time polymerase chain reaction was used for quantification of PEMT mRNA in lung cancer tissues. Lipoprotein lipase (LPL) and fatty acid synthase (FASN) activities had already been measured in the same tissues. During a four-year follow-up, 21 patients succumbed to tumor progression. One patient did not survive due to non-cancer reasons and was not included in the analysis. Cox regression analysis was used to assess the prognostic value of *PEMT* expression. Our findings show that elevated *PEMT* expression in the cancer tissue, relative to that in the adjacent non-cancer lung tissue, predicts shorter patient survival independently of standard prognostic factors and also independently of increased LPL or FASN activity, the two other lipid-related predictors of shorter patient survival. These findings suggest that active phosphatidylcholine and/or choline metabolism are essential for tumor growth and progression.

## Introduction

**Figure 1:**
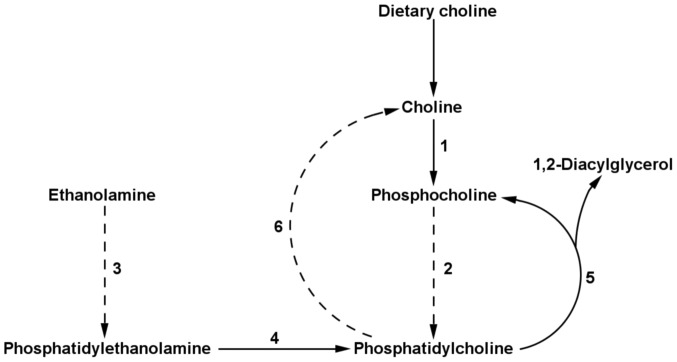
Pathways of PC metabolism. Enyzmatic reactions are indicated by numbers. Dashed lines present more reaction steps. 1 and 2, PC synthesis via the CDP-choline pathway; 3, the CDP-ethanolamine pathway of phosphatidylethanolamine synthesis; 4, PC synthesis by phosphatidylethanolamine N-methyltransferase; 5 and 6, PC degradation by various phospholipases and lysophospholipases. PC, phosphatidylcholine; CDP, cytidine diphosphate.

Phosphatidylcholine (PC), an essential phospholipid component of cell membranes, is synthesized in all nucleated cells via the cytidine diphosphate (CDP)-choline pathway (1), which requires the uptake of dietary choline. Alternatively, PC is synthesized by phosphatidylethanolamine N-methyltransferase (PEMT; EC 2.1.1.17), an enzyme responsible for the catalytic conversion of phosphatidylethanolamine (PE) to PC (Fig. 1) (2). PC synthesis via the PEMT pathway is dietary choline independent. *PEMT* expression and its activity are highest in the liver, where the PEMT pathway accounts for 30% of PC synthesis (1). PEMT plays an important role in hepatocytes. For example, *PEMT*^−^/^−^ mice had a decreased PC/PE ratio, which resulted in the loss of cell membrane integrity and liver failure (3). All other tissues exhibit low PEMT activity of, at most, 2% of the liver PEMT activity (4).

Highly proliferating cancer cells must continuously provide lipids for membrane assembly, signal transduction and protein modification (5). The biosynthetic and bioenergetic requirements for cell proliferation and survival are supported by vigorous fatty acid metabolism (6). Importantly, endogenously synthesized fatty acids are not stored as triglycerides, but are predominantly converted to phospholipids for membrane assembly (6,7). This may require active PC-synthesis pathways. PC concentration was found to be elevated in colorectal cancer (8,9) and a higher expression of the CDP-choline pathway enzymes was found in various types of human cancers (8,10). With regards to the PEMT pathway, data are inconsistent. Malignant neoplasm cells with metastases were characterized by a higher PC/PE ratio compared with malignant neoplasm cells without metastases (9), which suggests that higher PEMT activity may be associated with tumor aggressiveness. On the other hand, in rats with chemically induced hepatocarcinogenesis, *PEMT2* expression (an isoform exclusively located in mitochondrial membranes of hepatocytes) was found to be diminished in liver cancer lesions and, consistently, total PEMT activity was decreased during different stages of tumor progression (11). Thus, PEMT involvement in cancer requires further evaluation.

Fatty acid synthase (FASN; EC:2.3.1.85), the key enzyme involved in neoplastic lipogenesis (12), is the sole protein capable of *de novo* synthesis of long-chain fatty acids (7). As such, *FASN* is highly expressed in various types of human cancers and in several cancer types elevated *FASN* expression is linked to poor prognosis (12–17). Consistently, in patients with resectable non-small-cell lung cancer (NSCLC), higher FASN activity in the cancer tissues (relative to the adjacent non-cancer lung tissue) was associated with adverse outcomes and predicts shorter patient survival (18).

Lipoprotein lipase (LPL; EC:3.1.1.34) is another lipid-related enzyme associated with tumor growth. Following parenchymal synthesis, LPL is translocated to the luminal endothelial surface where it is responsible for intravascular catabolism of triglyceride-rich lipoproteins and extracellular supply of long-chain fatty acids, lipids and lipoproteins to adjacent tissues. Thus, patients with resectable NSCLC had a higher LPL activity in the cancer tissue than in the adjacent, healthy non-cancer lung tissue (19), and elevated LPL activity in resectable NSCLC tissue (relative to adjacent non-cancer lung tissue) predicts shorter patient survival independently of the standard prognostic factors (20).

To the best of our knowledge, PEMT has not yet been studied in NSCLC tissue in the literature to date and has not been studied concurrently with other lipid-related enzymes known to be associated with tumor growth, such as FASN or LPL. In order to study PEMT involvement in tumor growth, we hypothesized that *PEMT* expression is increased in NSCLC tissue and that increased gene expression acts as a predictor of shorter patient survival. *PEMT* expression was determined in the same NSCLC tissue samples as used for FASN and LPL analyses (18–20).

## Materials and methods

### Patient selection and tissue sampling

Forty-two patients (median age of 62.5 years) undergoing surgical removal of resectable, stages I, II and III NSCLC tissues at University Medical Centre Ljubljana (Ljubljana, Slovenia) were enrolled consecutively in this study. Samples of lung cancer tissues and of adjacent, visually unaffected tissues were cut from the resected lung within 15 min of surgery. Tumor tissues were taken from the periphery, where the tumor was most vigorous. Presumed normal tissues, taken from each subject as control, were cut from the periphery of the resected lung, far away from the tumor. Tissue samples were stored in liquid nitrogen until analyzed. Staging was performed according to the tumor-node-metastasis classification (21). Histological analysis of tumor tissue samples was performed in accordance with the World Health Organization histological classification (22). The study was approved by the National Ethics Committee and informed consent was obtained from all the patients participating in the study.

Patients were followed-up for four years. For the first two years, patients were examined every three months and in the second year, every six months. Clinical status and X-ray imaging results were assessed routinely at the time of examination. In progressively suspicious cases, bronchoscopy, computed tomography scans and scintigraphy were performed as required. None of the patients had undergone neoadjuvant therapy prior to surgery. Patients with stage III NSCLC underwent postoperative chemotherapy.

### Quantification of PEMT expression in tissue samples

RNA was isolated from tissue with RNeasy Mini kit (Qiagen, Hilden, Germany) and reverse transcribed to cDNA using the High-Capacity cDNA Archive kit (Applied Biosystems, Foster, CA, USA) as previously described (23).

SYBR Green-based real-time polymerase chain reaction (PCR) was used for the quantification of PEMT mRNA. A total of 2 μl cDNA, previously diluted with purified water (dilution ratio 1:5) (PCR Grade Water; Roche Diagnostics, Basel, Switzerland), was mixed with 0.5 μl of primer [PEMT or β-glucuronidase (GUSB) QuantiTect Primer Assays; Qiagen] and 2.5 μl FastStart Essential Green Master (Roche Diagnostics). Two separate analyses of all the samples were performed. In each analysis, all the reactions were run in duplicate. Reaction mixtures were incubated in a LightCycler^®^ 480 Instrument (Roche Diagnostics) for 10 min at 95°C, followed by 40 cycles of 15 sec denaturation at 95°C and 30 sec annealing/extension at 60°C.

GUSB was selected as the most appropriate internal control among the 11 candidates available on TaqMan Human Endogenous Control Plate (Applied Biosystems), as previously described (23). The average PEMT and GUSB PCR amplification efficiency (E) was considered in calculating relative PEMT mRNA quantities (RQ) using the following equation: RQ=(1+E) exp (−ΔΔCt).

### Statistical analysis

Data are presented as the medians with 25th and 75th percentiles and range values. Comparisons of parameters between cancer and control tissues were made with the Wilcoxon matched-pair test. Mann-Whitney or Pearson’s χ^2^ tests were used for comparisons of the same parameter between groups. Associations between variables were evaluated with Spearman rho analysis. Uni- and multivariate Cox regression analyses were used to assess the prognostic factors. Statistical analysis was performed with SPSS software, v.17.0 (SPSS Inc., Chicago, IL, USA). P<0.05 was considered to indicate a statistically significant difference.

## Results

During the follow-up, one patient did not survive due to non-cancer reasons and, therefore, was excluded from the statistical analysis. Baseline clinical characteristics of the remaining 41 patients are summarized in the first part of Table I. *PEMT* expression in the cancer tissues was equal to its expression in the adjacent non-cancer tissues and did not correlate with the disease stage (results not shown). Furthermore, in patients with squamous cell carcinoma, *PEMT* expression in the cancer tissue was equal to that in patients with adenocarcinoma (results not shown). During the four-year follow-up, 21 of the 41 patients succumbed to tumor progression with a mean survival time of 24.6±16.4 months.

For the survival analysis, *PEMT* expression was specified as the ratio of gene expression in cancer tissues to that in non-cancer tissues. Descriptive statistics of *PEMT* expression ratios are presented in the second part of Table I. Deceased patients had a 32% higher *PEMT* expression ratio in comparison with the patients that were alive four years after tumor excision; however, the difference was not significant (median, 1.135 vs. 0.0860; P=0.0556). *PEMT* expression ratio was negatively correlated with survival time following surgery, but this correlation was not statistically significant (Spearman’s ρ=−0.2745; P=0.0786). Cox-regression analysis revealed that increased *PEMT* expression ratio predicted shorter patient survival, independently of increased disease stage and weight loss, the two other predictors of shorter patient survival in univariate statistical analysis (Table II, Model 1).

In order to study *PEMT* involvement in tumor growth and progression concurrently with other lipid-related enzymes associated with tumor progression, such as FASN and LPL, FASN activity ratios (ratio of activity in cancer tissue to that in adjacent non-cancer tissue for each individual patient) and LPL activity ratios (ratio of activity in cancer tissue to that in adjacent non-cancer tissue for each individual patient) were used, which were estimated previously in the same tissues and shown to predict shorter patient survival (18–20). Descriptive statistics of FASN and LPL activity ratios are presented in the second part of Table I. *PEMT* expression ratio did not correlate with FASN and LPL activity ratios (results not shown) and increased *PEMT* expression ratio predicted shorter patient survival, independently of the increased FASN (Table II, Model 2) and LPL activity ratios (Table II, Model 3).

## Discussion

The present study shows that in patients with resectable NSCLC, elevated *PEMT* expression in the cancer tissue (relative to the adjacent non-cancer lung tissue) predicts shorter survival, independently of standard prognostic factors and also independently of increased FASN or LPL activities in the cancer tissue.

Notably, increased *PEMT* expression in lung cancer tissue is associated with adverse patient outcomes and is explicable by several potential causes. Current data regarding PEMT in cancer is scarce and controversial. On the one hand, malignant colorectal tumors have an increased PC/PE ratio (9), which suggests that increased PEMT activity is an essential mechanism for maintaining membrane integrity and to prevent cell death. Furthermore, *PEMT*^−^/^−^ mice fed with a choline-deficient diet did not develop hepatic cancer, but hepatic steatosis (24). On the other hand, in hepatocarcinomas, *PEMT2* expression and hepatic PEMT activity are decreased (11), and hepatocytes with overexpressed *PEMT2* are poorly tumorigenic (25). Furthermore, *PEMT* promoter polymorphism, −774G>C, which results in lower *PEMT* expression and choline deficiency (26), was associated with increased breast cancer risk among women receiving hormone replacement therapy (27). PEMT predictive capacity seems reasonable due to several causes. First, lipid mobilization is required to sustain tumor growth (28). Highly proliferating cancer cells use fatty acids (either synthesized endogenously by FASN or supplied exogenously from the microvascular system) predominantly for the synthesis of phospholipids, which are then incorporated in cell membranes (7). PEMT may play an important role in membrane assembly as PC, the end-product of the PEMT reaction, is the most abundant phospholipid in cell membranes. The CDP-choline pathway, which is the sole pathway for *de novo* synthesis of PC in non-hepatic cells, may not be sufficient for PC supply in cancer cells; therefore, cancer cells activate the PEMT pathway to cover the increased demand for PC to maintain an adequate PC/PE ratio, membrane integrity and cell survival. Furthermore, PC synthesis via the PEMT pathway is dietary choline independent. White adipose tissue covers its increased demand for new phospholipids, which are constituents of lipid droplets, by increasing *PEMT* expression (29). The second potential cause of PEMT predictive capacity may be its role in the only known endogenous pathway of choline synthesis, via the degradation of PC (Fig. 1) (30). The increased total level of choline-containing compounds has been found in several types of cancer (10) and the endogenous synthesis of choline appears to be upregulated in various cancer cells (31–35). Endogenous choline synthesis via the PEMT pathway may be essential particularly in the case of inadequate dietary choline uptake during increased cell requirements. Furthermore, choline is utilized as a methyl group donor and increased choline affects DNA methylation and results in disruption of DNA repair (36). The third potential cause of PEMT predictive capacity is that products of PC hydrolysis (phosphocholine and diacylglycerol) may function as secondary messengers (37) and certain enzymes involved in PC degradation (such as phospholipase D) may be implicated in cell proliferation, signal transduction, malignant transformation and tumor progression (36). Thus, PEMT may play an important role in tumor growth and progression in addition to lipid mobilization; however, these speculations require further investigation.

The finding that elevated *PEMT* expression in NSCLC tissue predicts shorter patient survival independently of increased FASN or LPL activities further strengthens the evidence that lipid mobilization is required to sustain tumor growth. Our previous studies showed that increased LPL and FASN activities are predictors of shorter survival in patients with NSCLC (18–20). PEMT predictive capacity in addition to LPL and FASN is explicable as FASN and LPL only supply the cancer tissue with long-chain fatty acids, which are then further used as constituents in phospholipid synthesis. PEMT is then involved in the synthesis of PC from PE, as cancer cells are characterized by increased growth and synthesis of new cell membranes has high requirements for PC. Moreover, other metabolic pathways associated with PEMT (such as endogenous choline synthesis, PC hydrolysis to secondary messengers and disruption of DNA repair) are also independent of long-chain fatty acid metabolism and, therefore independent of LPL and FASN activity levels. As both enzymes are molecular targets for cancer therapy, with specific inhibitors of FASN activity (7,38) or by inducing LPL activity in non-cancer tissues by peroxisome proliferator-activated receptor γ agonists (39,40), PEMT may also be a promising diagnostic and therapeutic target. Choline-containing compounds were found to be significantly elevated in prostate cancer versus benign prostate tissues, hence choline phospholipid metabolites can potentially be used for diagnosing and managing prostate cancer patients (36). In addition, it was reported that hexadecylphosphocholine, the alkylphospholipid analog, reduces cell proliferation in hepatoma cells and simultaneously inhibits PC synthesis via the CDP-choline and PEMT pathways (41).

In conclusion, increased *PEMT* expression in NSCLC tissues (relative to the adjacent non-cancer lung tissue) predicts shorter patient survival, independently of increased FASN or LPL activities in the same cancer tissues.

## Figures and Tables

**Table I:** Clinical characteristics and laboratory findings of patients with resectable non-small-cell lung cancer.

Parameters	Subjects
No.	41
Gender, female/male	11/30
Age, years	63.0 {53.0/67.0} (44–77)^a^
Body mass index, kg/m^2^	24.8 {22.2/28.3} (16.4–46.5)^a^
Weight loss in the last three months, no/yes	24/17
Smoking status, never/current or former	5/36
Tumor stage^b^, I/II/III	17/14/10
Histological type, squamous cell/adenocarcinoma/macrocellular/other	19/13/5/4
*PEMT* expression ratio (expression in lung cancer tissue vs. non-cancer lung tissue)	0.93 {0.59/1.22} (0.25–3.27)^a^
FASN activity ratio^c^ (activity in lung cancer tissue vs. non-cancer lung tissue)	1.48 {0.77/2.79} (0.04–54.07)^a^
LPL activity ratio^c^ (activity in lung cancer tissue vs. non-cancer lung tissue)	1.91 {1.08/2.42} (0.10–19.27)^a^

a Data are presented as: median {25th percentile/75th percentile} (minimum-maximum value).

b According to the tumor-node-metastasis classification (21).

c Estimated in the same tissues (18–20) as *PEMT* expression.

*PEMT*, gene encoding phosphatidylethanolamine N-methyltransferase; FASN, fatty acid synthase; LPL, lipoprotein lipase.

**Table II:** Predictors of shorter survival in patients with resectable non-small-cell lung cancer.

Model No.	Variables in the model (Cox regression analysis)	Relative risk (95% CI)	P-value
1	Disease stage (I vs. II or III)	5.87 (1.64–20.96)	0.006
	Weight loss in the last three months (no vs. yes)	3.83 (1.45–10.15)	0.007
	*PEMT* expression ratio (low vs. high)^a^	3.55 (1.35–9.33)	0.010
2	Disease stage (I vs. II or III)	4.42 (1.23–15.95)	0.023
	Weight loss in the last three months (no vs. yes)	3.50 (1.27–9.70)	0.016
	*PEMT* expression ratio (low vs. high)^a^	3.08 (1.16–8.15)	0.023
	FASN activity ratio (low vs. high)^a^,^b^	2.36 (0.85–6.57)	0.101
3	Disease stage (I vs. II or III)	3.39 (0.94–12.24)	0.063
	Weight loss in the last three months (no vs. yes)	2.51 (0.88–7.15)	0.084
	*PEMT* expression ratio (low vs. high)^a^	3.00 (1.09–8.25)	0.033
	LPL activity ratio (low vs. high)^a^,^c^	4.18 (1.26–13.87)	0.019

a Below or above the median value of the analyzed variable.

b Estimated in the same patients previously (18).

c Estimated in the same patients previously (19,20).

*PEMT*, gene encoding phosphatidylethanolamine N-methyltransferase; FASN, fatty acid synthase; LPL, lipoprotein lipase.

## References

[1] Shields DJ, Agellon LB, Vance DE (2001). Structure, expression profile and alternative processing of the human phosphatidylethanolamine N-methyltransferase (*PEMT*) gene. Biochim Biophys Acta.

[2] Vance DE, Li Z, Jacobs RL (2007). Hepatic phosphatidylethanolamine N-methyltransferase, unexpected roles in animal biochemistry and physiology. J Biol Chem.

[3] Li Z, Agellon LB, Allen TM (2006). The ratio of phosphatidylcholine to phosphatidylethanolamine influences membrane integrity and steatohepatitis. Cell Metab.

[4] Vance DE, Vance JE, Vance DE, Vance JE (2008). Phospholipid biosynthesis in eukaryotes. Biochemistry of lipids, lipoproteins and membranes.

[5] Bauer DE, Hatzivassiliou G, Zhao F, Andreadis C, Thompson CB (2005). ATP citrate lyase is an important component of cell growth and transformation. Oncogene.

[6] Barger JF, Plas DR (2010). Balancing biosynthesis and bioenergetics: metabolic programs in oncogenesis. Endocr Relat Cancer.

[7] Kuhajda FP (2006). Fatty acid synthase and cancer: new application of an old pathway. Cancer Res.

[8] Dueck DA, Chan M, Tran K (1996). The modulation of choline phosphoglyceride metabolism in human colon cancer. Mol Cell Biochem.

[9] Dobrzyńska I, Szachowicz-Petelska B, Sulkowski S, Figaszewski Z (2005). Changes in electric charge and phospholipids composition in human colorectal cancer cells. Mol Cell Biochem.

[10] Glunde K, Bhujwalla ZM, Ronen SM (2011). Choline metabolism in malignant transformation. Nat Rev Cancer.

[11] Tessitore L, Dianzani I, Cui Z, Vance DE (1999). Diminished expression of phosphatidylethanolamine N-methyltransferase 2 during hepatocarcinogenesis. Biochem J.

[12] Flavin R, Peluso S, Nguyen PL, Loda M (2010). Fatty acid synthase as a potential therapeutic target in cancer. Future Oncol.

[13] Ogino S, Nosho K, Meyerhardt JA (2008). Cohort study of fatty acid synthase expression and patient survival in colon cancer. J Clin Oncol.

[14] Rossi S, Graner E, Febbo P (2003). Fatty acid synthase expression defines distinct molecular signatures in prostate cancer. Mol Cancer Res.

[15] Shurbaji MS, Kalbfleisch JH, Thurmond TS (1996). Immunohistochemical detection of a fatty acid synthase (OA-519) as a predictor of progression of prostate cancer. Hum Pathol.

[16] Takahiro T, Shinichi K, Toshimitsu S (2003). Expression of fatty acid synthase as a prognostic indicator in soft tissue sarcomas. Clin Cancer Res.

[17] Visca P, Sebastiani V, Botti C (2004). Fatty acid synthase (FAS) is a marker of increased risk of recurrence in lung carcinoma. Anticancer Res.

[18] Cerne D, Zitnik IP, Sok M (2010). Increased fatty acid synthase activity in non-small cell lung cancer tissue is a weaker predictor of shorter patient survival than increased lipoprotein lipase activity. Arch Med Res.

[19] Cerne D, Melkic E, Trost Z, Sok M, Marc J (2007). Lipoprotein lipase activity and gene expression in lung cancer and in adjacent noncancer lung tissue. Exp Lung Res.

[20] Trost Z, Sok M, Marc J, Cerne D (2009). Increased lipoprotein lipase activity in non-small cell lung cancer tissue predicts shorter patient survival. Arch Med Res.

[21] Sobin LH, Wittekind C (2002). Lung and pleural tumours. TNM Classification of Malignant Tumours.

[22] Travis WD, Brambilla E, Muller-Hermelink HK, Harris CC (2004). World Health Organization Classification of Tumours. Pathology and Genetics of Tumours of the Lung, Pleura, Thymus and Heart.

[23] Trost Z, Marc J, Sok M, Cerne D (2008). Increased apolipoprotein E gene expression and protein concentration in lung cancer tissue do not contribute to the clinical assessment of non-small cell lung cancer patients. Arch Med Res.

[24] Vance JE, Vance DE (2005). Metabolic insights into phospholipid function using gene-targeted mice. J Biol Chem.

[25] Tessitore L, Sesca E, Vance DE (2000). Inactivation of phosphatidylethanolamine N-methyltransferase-2 in aflatoxin-induced liver cancer and partial reversion of the neoplastic phenotype by PEMT transfection of hepatoma cells. Int J Cancer.

[26] da Costa KA, Kozyreva OG, Song J (2006). Common genetic polymorphisms affect the human requirement for the nutrient choline. FASEB J.

[27] Xu X, Gammon MD, Ziesel SH (2008). Choline metabolism and risk of breast cancer in a population-based study. FASEB J.

[28] Mulligan HD, Tisdale MJ (1991). Effect of the lipid-lowering agent bezafibrate on tumour growth rate *in vivo*. Br J Cancer.

[29] Hörl G, Wagner A, Cole LK (2011). Sequential synthesis and methylation of phosphatidylethanolamine promote lipid droplet biosynthesis and stability in tissue culture *in vivo*. J Biol Chem.

[30] Li Z, Vance DE (2008). Phosphatidylcholine and choline homeostasis. J Lip Res.

[31] Qi C, Park JH, Gibbs TC (1998). Lysophosphatidic acid stimulates phospholipase D activity and cell proliferation in PC-3 human prostate cancer cells. J Cell Physiol.

[32] Foster DA, Xu L (2003). Phospholipase D in cell proliferation and cancer. Mol Cancer Res.

[33] Stewart JD, Marchan R, Lesjak MS (2012). Choline-releasing glycerophosphodiesterase EDI3 drives tumor cell migration and metastasis. Proc Natl Acad Sci USA.

[34] Cao MD, Döpkens M, Krishnamachary B (2012). Glycerophosphodiester phosphodiesterase domain containing 5 (GDPD5) expression correlates with malignant choline phospholipid metabolite profiles in human breast cancer. NMR Biomed.

[35] Kang DW, Choi KY, Min do S (2011). Phospholipase D meets Wnt signaling: a new target for cancer therapy. Cancer Res.

[36] Awwad HM, Geisel J, Obeid R (2012). The role of choline in prostate cancer. Clin Biochem.

[37] Ackerstaff E, Glunde K, Bhujwalla ZM (2003). Choline phospholipid metabolism: a target in cancer cells?. J Cell Biochem.

[38] Mashima T, Seimiya H, Tsuruo T (2009). De novo fatty-acid synthesis and related pathways as molecular targets for cancer therapy. Br J Cancer.

[39] Nomura K, Noguchi Y, Matsumoto A (1996). Stimulation of decreased lipoprotein lipase activity in the tumour-bearing state by the antihyperlipidemic drug bezafibrate. Surg Today.

[40] Mutoh M, Niho N, Wakabayashi K (2006). Concomitant suppression of hyperlipidemia and intestinal polyp formation by increasing lipoprotein lipase activity in Apc-deficient mice. Biol Chem.

[41] Jiménez-López JM, Carrasco MP, Segovia JL, Marco C (2004). Hexadecylphosphocholine inhibits phosphatidylcholine synthesis via both the methylation of phosphatidylethanolamine and CDP-choline pathways in HepG2 cells. Int J Biochem Cell Biol.

